# Neighborhood-resources for the development of a strong SOC and the importance of understanding why and how resources work: a grounded theory approach

**DOI:** 10.1186/s12889-017-4705-x

**Published:** 2017-09-12

**Authors:** Ruca Maass, Bengt Lindström, Monica Lillefjell

**Affiliations:** 1 Department of Neuromedicine and Movement Science, Faculty of Medicine and Health Sciences, Norwegian University of Science and Technology, Trondheim, Norway; 2 NTNU Center for Health Promotion Research, Norwegian University of Science and Technology, Trondheim, Norway; 3 Departement of Public health and Nursing, Faculty of Medicine and Health Sciences, Norwegian University of Science and Technology, Trondheim, Norway

**Keywords:** Sense of coherence (SOC), Health promotion, Neighborhood, Generalized resistance resources (GRRs), Salutogenesis, Focus group research, Grounded theory, Public health

## Abstract

**Background:**

Providing individuals with psychosocial resources such as sense of coherence (SOC) seems a beneficial strategy for health promotion in the neighborhood. In order to become a supporting theory for health promotion, Salutogenesis should renew its focus on resources for health, and explore how the development of a strong SOC can be facilitated.

**Methods:**

Relevant issues were explored using a Grounded Theory- approach. Three focus-group-sessions and three in-depth interviews were conducted with strategically sampled participants. The transcripts of the focus groups were initially analyzed line-by-line to ensure that insights emerged from the data. We then applied focused and systemic analyses to achieve axial coding, and to include insights into how social interactions during focus groups may reveal social processes in real-life-neighborhoods. The data from the in-depth interviews were used to validate and fill emerging categories, as well as to ensure data-saturation.

**Results:**

Findings indicate the importance of repeated experiences with resources and every-day-challenges to develop a strong SOC. Active engagement with resources is a favorable condition for significant experiences, which enhance the internalization of resources. Core experiences are characterized by a re-organization of resources. Participation in intellectual meaning-making through equal power dialogue seems to broaden perspectives and promote the strengthening of SOC. A strong SOC can also be described as a deeper understanding of how and why resources work, which allows for a more flexible use of resources, including replacing missing resources.

**Conclusion:**

A new understanding of SOC as an intuitive understanding of how, why and under which circumstances resources work, as well as a new focus on everyday life and repeated experiences might facilitate new approaches to a purposeful strengthening of SOC through the planning and implementation of public measures.

## Background

A growing body of research examines how neighborhoods can contribute to the promotion of health for their inhabitants. According to the WHO’s Ottawa-charter [1] and the Shanghai-declaration on Health Promotion [2], local communities and neighborhoods are central arenas for health promotion (HP) efforts. This requires more knowledge about how public measures can contribute to making neighborhoods beneficial HP- arenas [3]. Neighborhoods are understood as super-settings, which are experienced on the base of internal micro-systems (e.g. families, social groups, organizations and institutions), and their inter-relations [4].

Reducing inequalities in health is a major goal in HP [5]. Health inequalities between neighborhoods have been linked to socio-economic status (SES), and highlight aspects of social justice and the social determinants of health (SDoH) [5–8]. Social injustice in health is reinforced through the distribution of resources and risks between and within neighborhoods, as well as through possibilities to influence decisions on a local level [9]. A number of factors- including social capital, belonging, access to green-space, walkability, quality of environmental resources, accessible and trustworthy institutions and inclusive planning strategies- have been linked to better health-outcomes [10–18]. In line with a health in all policies (HiAP)-approach, HP in the neighborhood seeks to improve conditions for daily living, provide resources, limit risk factors, and reduce inequalities in and between neighborhoods, including power-distributions [5, 7, 9]. Research suggests that HP in neighborhoods should aim at equipping individuals with psychosocial, emotional and social resources, such as Sense of coherence (SOC) [18, 19].

SOC is an important concept within Salutogenesis. It describes lasting perceptions of the world as comprehensive, manageable, and meaningful [20]. In Salutogenesis, health is experienced along a continuum that ranges from “total health” to “un-health”. The focus is on resources and assets for health [21]. Antonovsky [20, 21] focuses on SOC as an individual concept, while he acknowledges that SOC can characterize any unit, including neighborhoods, cities, and regions [21]. He points out that group-SOC should be measured independently from individual SOC ([20]: p.172). Our focus is on individual SOC, and how it is shaped by neighborhood- and community-factors.

SOC is developed through the internalization of resources [20–22]. The internalization-process turns resources into “generalized resistance resources” (GRRs), and is driven by so-called significant life events [20]. Life events can affect SOC positively or negatively, dependent on available resources: if sufficient GRRs are available, challenges are resolved, and SOC is strengthened. If GRRs are lacking or inappropriate, challenges remain un-resolved or are resolved poorly, and SOC is weakened. Moreover, a strong SOC can also been described as the ability to easily identify and adequately use resources ([18, 21]: p.132, [22]). For example, among adolescents with congenital heart disease, strong-SOC participants reported as much negative life events as weak-SOC participants, but also more available resources [23]. Partly, this could be explained by an increased ability to identify resources. The relationship between resources and SOC is circular: resources help to solve challenges and strengthen SOC, and a strong SOC helps to find and adequately use resources, resolve challenges and thereby again strengthen SOC [20].

While conducting this study, we became aware of how the relationship between user and resource can facilitate more or less significant experiences: In ‘passive’ relationships, resources are instrumentalized to achieve life goals (small or large). In ‘active’ relationships, participants engage with and enter into a dialogue with the resource itself. Antonovsky’s starting point was resources for health and GRRs [21]. Since then, most of the salutogenic research has focused on the link between SOC and favorable health-outcomes, including aspects of Quality of Life [24, 25]. However, to become a beneficial theory for HP, Salutogenesis should include questions about how to facilitate the development of a strong SOC [19, 26]. This is in line with Antonovsky’s claim that investigating conditions that shape a strong SOC would be a central issue for future research [20].

Societal conditions, including power distributions, shape the SOC by providing individuals with repeated, systematic experiences [21]. Research shows that the neighborhood is an important arena for the development of SOC, behind family and school-context [26]. Neighborhood cohesion, social support and informal control over adolescents are linked to a strong SOC [27, 28]. Satisfaction with accessibility and quality of physical and structural neighborhood-resources, as well as social capital and overall thriving show significant links to SOC-scores across social groups [18].

The focus of this paper is how neighborhoods can facilitate for the development of a strong SOC. Neighborhoods are understood as super-settings or open systems. Public measures and resources can thereby influence experiences in and with the local environment [4]. Accordingly, the focus is on directly influence-able, material/physical (sports-halls, community-houses, green-spaces etc) and structural resources (transportation, public institutions, information, influence). How can these contribute to perceptions of the world as comprehensive, manageable and meaningful?

## Methods

This study is a qualitative follow-up of a quantitative population survey conducted in the municipality of Malvik in 2012 [29]. The data were gathered through focus groups and in-depth interviews, and analyzed using an adapted Grounded Theory approach [30]. To ensure that we understand how information is produced through social processes visible during the focus groups, elements of systemic analysis are included into the later steps-of-analysis [31]. Categories are developed based on findings from the focus groups, as well as the conceptual model of SOC. They were then applied back on the transcripts from both focus groups and in-depth interviews, to deepen insights through triangulation.

### Focus groups: Participants and composition

Three focus groups with between three and eight participants were conducted. The participants lived in one of the three census-tracts in which the population-survey had been distributed. Recruitment-strategies included calls for participation on the municipality’s web- and Facebook-pages, flyers distributed to inhabitants’ mailboxes, and an article published in the local newspaper. Eleven women and eight men participated, their (estimated) age ranging from mid-thirties to late-sixties. Most of the participants had lived in their present neighborhood for more than five years.

According to our applied focus is the neighborhood-of-residency an important level-of-analysis. Therefore, the participants were matched according to their area-of-living: Focus groups were composed of participants from two approximate neighborhoods within a common census-tract. This was considered to reveal insights into social processes within and between neighborhoods, which might influence how resources are perceived and used [32].

We applied a semi-structured interview-guide, including topics such as health-relevant resources, positive factors and general thoughts on the health-impact of neighborhoods, reflections about why something might work as a resource, and questions about experiences in the neighborhood that changed the participants’ outlook on the world.

### In-depth interviews

After the initial analysis of the focus-group-transcripts, three in-depth interviews were conducted. The informants were sampled strategically: two of them were younger (mid-twenties) than the former participants. The last one was chronically ill, and served as a member of the municipal advisory board for people with disabilities and chronic sickness. These participants added valuable perspectives from new social groups, and were considered to enrich the collected data. A slightly modified version of the interview-guide was applied. In-depth interviews were mainly used to confirm, and fill emerging categories with content. Moreover, in-depth interviews indicated data-saturation: no all-new topics emerged during the in-depth interviews [31].

### Analysis strategy

All of the interviews were transcribed completely. To keep track of individuals during the analysis, every participant was assigned a number that reflected their gender, focus group and neighborhood. However, to ensure the anonymity of the participants who live in small local communities, we decided not to identify the participants in any way in this article. Instead, participants (P) are numbered in the chronological order in which they enter the dialogue separately for each citation.

We began with a line-by-line-analysis as described by Charmaz [30]. Then, we applied focused coding to merge emerging categories with the theoretical background of Salutogenesis and the conceptual model of SOC, including its three dimensions (comprehensibility, manageability and meaningfulness) [20, 21, 30]. Axial coding was achieved through constant comparison [30, 31]. Thereby, the theoretical framework linking external resources to SOC-development was established.

Sampling heterogeneous groups can provide insights into how understandings are created within groups [32, 33]. As described above, we matched real-life neighbors from two approximate neighborhoods into focus groups to achieve insights into social processes occurring in and between real-life neighborhoods. The data from the focus groups were analyzed on two levels: what was said, and what happened during focus groups. The possibility- and demand- to regard social processes within the focus group is considered to be one of the most important aspects of focus-group research [33, 34]. As this requires the analysis of longer passages and conversations, and understanding information in the context of the social interaction in which it is produced, we applied focused coding, as well as elements of systemic analysis: Initial codes were translated into broader categories, and embedded in the inter-relationships between these categories. This was done to make the social processes visible, and accessible for analysis [32].

To ensure that concepts emerged from the data, we took a step back from the theoretical background after having established an initial understanding. A lot of effort was put into understanding what was going on and linking these insights to the conceptual model without being led by theoretical pre-conceptions. Only after having concluded the empirical analysis, did we return to the original sources [20, 21] to consider how findings fit the theoretical framework, and if and where we could make new contributions to the emerging field of salutogenic HP- research.

As the data from the focus groups are considered the main body of data, the above-described steps were mainly applied to the focus-group-transcripts. The data from the in-depth interviews was used to confirm emerging concepts, to fill earlier-developed categories with content, and to deepen insights through triangulation [30].

### Ethics, consent and permissions

All research-participation is a real-life-event, especially when conducting research in real-life-settings, as gathering real-life neighbors into focus groups. HP-research should seek to reduce the risk, and to promote the benefits of participation. To protect privacy and to reduce risk during and after participation, we avoided too-personal questions during group-sessions, and strictly followed procedures and ethical guidelines regarding anonymity, including not identifying individuals in this text. Every participant signed an informed consent form before participation, and was given the opportunity to withdraw consent after the focus group. This study was approved by the regional ethical committee on medical research (REK).

## Results and discussion

### Neighborhoods, health-relevant resources and SOC

Participants identified health-relevant resources found at different levels of experience. Material resources included natural sites, such as access to the seaside and green-spaces, the surrounding landscape, and characteristics such as “small” and “transparent”. Participants listed amenities and institutions run by the municipality, commercial stakeholders or locals, such as sports-arenas, shopping- opportunities, cultural activities, or community-houses.

Structural resources included public transportation, bike- and walkable pathways, information, activities and resources, channels of communication and possibilities for participation in decision-making. Regular events, such as annual celebrations or shared efforts (Norwegian ‘*Dugnad*’), were also described as resources, and linked to social bonding and sense-of-place.

Social and psychological resources included “*good neighbors*”, belonging to the social community or the place as such, and feeling safe. A *“good neighborhood”* implied a certain degree of mutual, as well as shared responsibility for the neighborhood. Such resources were linked to material and structural resources, and described as desired outcomes. Accordingly, the focus of this article is on material and structural resources, as they can be influenced directly.

Gathering participants from two different neighborhoods highlighted matters of local context: what worked as a resource in one neighborhood did not work in others. This was for example illustrated by descriptions of a community-house (located in neighborhood A):

P1: “*This is our barrel of gold, this house. Our most important thing. To make people come together. It’s convenient, and much easier to accomplish stuff locally”*

P2: *“It’s possible to have celebrations here, weddings, confirmations, birthdays…”*

P3: *“Training, play-club, youth-club, senior-café, Christmas-parties, even Zumba”*

In the other participating neighborhood (B) was a similar house, owned by the local yacht club. However, inhabitants told that it’s use was limited to be rented for general assemblies or big celebrations. In contrast to the house in neighborhood A, a participant from neighborhood B reflected that “*I would not point [the house] out as health-promoting in any way”.* Clearly, context influenced whether a feature was perceived as a resource. According to Salutogenesis, a resource is defined by it’s ability to facilitate good tension-management [21]. During their life-course do individuals encounter times of tension which may- or may not- be resolved with the external or internal resources at hand. Thus, resources and stressors are two sides of the same coin, partly depending on contextual matters [21]. This duality became visible during focus-groups:

P: “*you do not need to go far before you find villages that have not taken care of their houses. And then it becomes a thing that costs you, and nobody feels it’s their own. So then it stands there and crumbles.”*

Thus, trying to achieve HP by placing resources in neighborhoods implies some risk: what is thought to be a resource, may instead become a stressor [34].

#### SOC

SOC became visible as the belief of being able to meet, and cope with challenges in life, either individually or collectively. This is in line with Antonovsky’s original definition of SOC as the “*confidence that one’s internal and external environments are predictable, and that (…) things will work out as good as could reasonably be expected*” ([21]: p.10). SOC also seems to imply a certain responsibility to take matters into one’s own hands. This responsibility to “*make things happen*” is linked to an internal locus of control [35]. However, according to Antonovsky, the important experience is “*things are under control*”, thereby including things under the control of peers or legitimate others [20]. Collectively, such perceptions were linked to perceived independence from the “*greater society*”. In terms of Salutogenesis, this can be described the ability to understand challenges, visualize solutions, be able to act on these visions, and to perceive it worthwhile to engage with the challenge- in other words, a strong SOC [36].

Negative impacts on SOC became visible in the context of major changes in the neighborhood, for example planned expansions: such times were described as “*confusing*”, “*not making sense*” and “*meaningless*”. Fundamental changes in the context seemed to threaten SOC. According to theory, SOC is stable throughout adulthood, even if life events can affect it temporarily and become visible as fluctuations around a stable mean [20]. Recent research suggests that SOC is not as stable as assumed, but increases over the whole life-span [37, 38]. Additionally, the stronger the SOC, the more stable it is: strong-SOC individuals are more likely to seek out and overcome challenges than weak-SOC individuals. For weak-SOC individuals, negative experiences can have lasting, negative effects on SOC [39].

#### How can external resources contribute to the development of a strong SOC?

The focus-group-sessions revealed how experiences were generalized, and influenced participants’ coping strategies and outlook on the world:

P: “*Well, I grew up in the West (…) and I climbed a lot. And there’s something about reaching a peak that you never managed before- I remember that feeling. And I still can recall all the cracks I used, even after 45 years. You learn that even if you don’t succeed the first time, you might if you try some more times, and maybe adapt what you are doing a little. And I can still recall the experience, you get rid of all the thoughts, and just concentrate (…) you train your concentration, and you remember the details, the way to handle a problem”*

This illustrates that even singular experiences can make lasting contributions to how we meet the world. One might wonder whether this describes a “*significant life events*” as anticipated in Salutogenesis ([21]: p.176). Prior research has described significant life events as major changes in life, for example the loss of loved ones, or going on a first long journey [40]. The above example does not fit into such descriptions of overwhelming events. Nevertheless, it clearly represented a transformative experience for the participant, at least in retrospect. Antonovsky acknowledges that any type of experience, even internal events, can become significant, and that the meaning of stressors emerges after the challenge is resolved-or not [21]. Accordingly, the above example may describe a spontaneous, internal transformation, facilitated by the engagement with a neighborhood-resource (the climbing-site) which represents both the challenge and (part of) the solution.

Partly, the significance of the experience might be due to it being a (inherently stressful) first-time-experience (“*you never managed before*”) ([21]: p.85f): responses to familiar stressors are by large automatic. New experiences make us draw on hitherto unfamiliar resources, or re-try familiar resources for new purposes. Antonovsky describes this as “*potentiation*”, as it is about exploring potential resources ([21]: p.96). However, *“going climbing”* is simultaneously described as a regular activity. It may thereby be a “SOC-reinforcing” experience sought out by persons with a strong SOC [21, 37]. Together with Antonvoksy’s focus on *repeated* (rather than *significant*) experiences, this points towards repetition as an important condition for internalization of resources [20].

Findings suggest that repeated use depends on the resource’s relevance, its ability to help solve challenges, and experiences of meaningfulness. Accordingly, the role of resources could change throughout the life-course: For example, a playground in neighborhood (C) was described as a formerly important arena, which was maintained by the local community. However, it lost its relevance when children grew up, and the challenge of fixing it up no longer seemed worthwhile [41]. This highlights the importance of the relationships between participant and resources. Recall the tale of the two community houses in neighborhood A and B, the first (A) being described as an omnipotent facilitator of shared activity. It was actively used, run and administered by locals, who thereby engaged with the resource as such. On the other hand, the second house (B) could be instrumentalized for specific purposes, but without participants engaging in it otherwise. The house was a tool for the experience, rather than a part of it: the relationship remained a passive one.

### Passive relationships between user and resources

‘Passive’ relationships describe relationships in which resources are used to achieve life goals (small or large), but during which the user does not engage with the resource as such. The resource is instrumentalized for specific purposes, but it is not perceived to be meaningful in its own right. Passive relationships were linked to achieving load-balance during everyday life [20]:

P: “*What makes it very ok to live here is that it is quite central, but at the same time, you can withdraw into some green areas (…) whatever you want, you can find it somewhere near. It is the sum of all these small things- that it is so convenient.”*

Matters of infrastructure, transportation, access to public services, sports-facilities and natural sites were mentioned explicitly. Matters of load-balance also affected choice of resources: Resources that were difficult to access or handle were dismissed, and replaced with other resources. For example, teenagers could prefer resources in the near-by cities over local ones because of lacking internal transportation. This could even affect serious decisions, such as choice of secondary school.

#### Local sources of information

The first step in using resources was to know about them. Being able to identify resources is linked to comprehensibility [20]. A lack of local information could be an obstacle for participation, and lead to difficulties with “*finding the way in*”, especially for new-comers.

Somehow unexpected did social media emerge as a little appropriate channel for spreading information, as they distribute information based on earlier actions (such as liking similar pages). Thus, information was primarily accessible to those who were already ‘in’. However, information-spreaders were not aware of this, but believed they were using inclusive communication-channels. Participants emphasized the importance of locally distributed, free information. A non-commercial neighborhood-newspaper was mentioned as resource in this regard, which simultaneously served as a mean for developing a local identity. A strong SOC enables people to easily identify resources [20]. Does this imply that easily identifiable resources and comprehensive, easily accessible information can diminish the importance of SOC for finding adequate resources?

Moreover, findings suggest that involvement in any neighborhood-arena increased comprehensibility about the neighborhood as such- partly through better knowledge about how to obtain information. This is in line with Antonovsky’s claim that being involved in one place increases knowledge about other societal arenas [21]. All in all, findings suggest that easily accessible information is crucial for comprehensibility. Lacking information makes it difficult to identify resources, and thereby to start participating, and make SOC-strengthening experiences [20].

#### Major changes in the neighborhood

Times of major changes in a neighborhood were associated with feelings of confusion and meaninglessness. Participants linked these feelings to unclear, mixed or lacking information about what was going to happen- in other words, the challenge was not fully understood [39]. Consistency between information and experience was important: Contradicting or confusing information, such as claiming to apply inclusive processes without doing so, seemed to affect meaningfulness as well as comprehensibility. What did not make sense intellectually did not make sense emotionally either [22]. Matters of meaningfulness are linked to ‘participation in decision-making’ and ‘emotional closeness’. The latter has not been verified empirically [42], but became clearly visible in this study:

P: *“There is so much good about this neighborhood, it just talks to your soul (…) it just makes me feel I belong; that this is home for me. This quietness when I walk home from the bus stop, it is just a fantastic feeling- like, now I am finally home in my own little realm”*

Being unable to understand or cope with anticipated changes in the neighborhood threatened feelings of belonging and emotional closeness by challenging perceived control over the neighborhood. Thus, it seems like perceptions of meaningfulness are indeed linked to ‘participating in shaping outcomes’. On the other hand, while passive relationships with resources seem to be able to facilitate for meaningful experiences and relationships, such resources were easily replaced if they were lost or altered. The ability to identify adequate replacements, and to hold flexible boundaries of what is important in life have been linked to a strong SOC ([20]: p.24). For example, being able to dismiss the playground during times of little relevance might be an expression of a strong SOC.

Moreover, it seems reasonable to assume that the above-described contributions to manageability and comprehensibility are somehow partial. They are derived from instrumentalizing resources for specific goals, and are linked to relevance rather than meaningfulness. Does this imply that they are too closely linked to specific situations to be considered GRRs? Increasing one or two, but not all dimensions of SOC might result in an un-stable SOC ([20]: p.21), or even in a ‘fake’ or ‘rigid’ SOC based on few and non-flexible strategies ([20]: p.25f). One might have comprehensive understandings of a specific challenge and how it could be solved. However, if this understanding remains linked to the specific challenge or setting, it does not build on ‘real’ comprehensiveness. This implies that instrumentalizing resources might contribute to the promotion of health, but the (individually perceived) generalizability of the resource may stay limited in this course. Might ‘passive relationships’ imply that the resource remains a specific resource (SSR)?

Moreover, while the playground was relevant for every-day life, did the participants have a rather active relationship with it. Findings indicate that active relationships are more closely linked to experiences of meaningfulness than passive ones. Partly, this might be due to including ‘participating in shaping outcomes’ in the applied definition of ‘meaningfulness’. However, it is possible that experiencing meaning in a given context facilitates for entering into an active dialogue with the context. As meaningfulness is described as “*most crucial component*” of SOC ([20]: p.22), this might be crucial for HP-strategies. Can we facilitate for experiences of meaningfulness by promoting active relationships with resources?

### Active relationships between user and resources

In “active” relationships, participants entered a dialogue, and engaged with the resource itself: either in terms of mastery, learning new ways to handle the resource, influence or adapt it, or experience meaningfulness during engagement. The resource became an integrated part of the experience, and was perceived to be meaningful in itself. Recall the climbing-example in which the participant describes experiences of flow and meaningfulness while actively engaging with a resource [43].

This also illustrates the importance of preventing under-load [20], and offering manageable challenges to strengthen SOC. By learning new ways of climbing the mountain, the participant increases climbing skills (manageability), forms a new understanding of how to take on challenges (comprehensibility), and experiences meaningfulness while doing so. Thus, the experience touches all three dimensions of SOC simultaneously, which, according to Antonovsky, is an important characteristic of a GRR [20]. However, our findings suggests that it is not the resource itself, but the active engagement with the resource that generates such experiences. According to theory, it might not be the event itself, but the experiences made in its wake that influence SOC [20]. Does this imply that significant life events trigger experiences touching into all three dimensions of SOC?

#### Active participation and comprehensibility: The importance of social interactions

Above, we discussed how “identifying resources” depends on available information. However, findings also suggest that social interactions play a prominent role in the process of identifying resources. Shared understandings about neighborhood-resources were established during focus groups: what are the resources, why they are resources, and for whom. In this process, participants actively sought after, and provided each other with confirmation: after an initial statement, they would wait for others’ responses before elevating their point-of-view. This highlights the need of the second level-of-analysis, and illustrates how meaning is made during focus groups [43, 44].

Responses often supported the initial statement, but could also provide additional, and even conflicting information. For example, one participant described her neighborhood as a tightly-knit community with many positive social encounters. This view was challenged by other participants, who perceived the neighborhood as little welcoming. However, the challenge was not met by ‘conflict’. Instead, the first participant acknowledged that the other description also might *“have some truth to it”*, as the neighborhood might seem hard to access for ‘newcomers’. By including different perspectives (‘newcomers’ versus ‘oldies’), participants were able to establish shared understandings of *how it can be.* Instead of relying solely on their own experiences, participants could thereby use others’ experiences actively to gain a more differentiated and comprehensive picture. Thus, participants improved their understanding about the resource through part-taking in active meaning-making- including why this is a resources, and how contextual features such as group-belonging can enhance or spoil its role as resource [18].

Antonovsky links comprehensibility to predictability, clarity of messages, strong values and structured surroundings [20]. However, these links have not been validated empirically, which has been explained by conflicts between receiving clear messages, and participating in decision-making as linked to meaningfulness [42]. Our findings offer an alternative explanation indicating that intellectual meaning-making may in itself be a more active process than anticipated, and can be spoiled by strict rules and clear, un-negotiable messages. Maybe increased comprehensibility it is not so much about discovering structure, but about participation in the creation of this structure?

#### Discovering structure and participation in decision-making

Including matters of power into the analysis helps to unravel these contradictory findings. Above, the municipality has the power to define both the outcome of, and the rules during the planning process. Thus, clear and consistent messages ‘from above’ are important for making sense of planned changes. The rules are ‘fixed’, and the opportunity to ‘discover structure’ increases comprehensibility and enables participation ([20]: p.96). Mixed messages and unpredictability lead to confusion and overload, and may weaken SOC in the long run.

This is illustrated by one participant’s attempt to influence a planned expansion in his neighborhood. He mobilized external resources and used existing channels-of-influence (sending a formal complaint and writing a letter to the local newspaper). However, he felt deliberately alienated by the municipality’s reaction, where they described his efforts as involvement by *“someone from the outside”.* The challenge was not resolved, and the experiences he made reinforced perceptions of incomprehensibility and overload, and threatened meaningfulness. This also changed his experiences with the resource:

P: *“I cannot relax in this beautiful green-space anymore, because I think about how long it’s going to last- and how it will be around there in a year or two. (…) This thought sabotages the experience. So, I prefer to go up to the lake, because I know there is small chance that it is going to be destroyed. If I want to really relax.”*

The participant coped by finding new resources to replace the lost one: being aware of it’s role and function in his life, he was able to identify another resource serving similar purposes. In a way, he found a satisfying solution, but this solution implied the loss of a resource, and changed his perceptions of his place in the municipality. Putting feedback into the setting is an important pre-requisite for strengthening SOC [20]: Participation in the process of “*making order*” can reveal meaningful information about how you can influence the arising structure. This includes knowledge about how the resource can help you to achieve desired outcomes ([20]: p.96).

Moreover, findings indicate that being involved in decision-making on a higher level could broaden perspectives, and enhance comprehensibility for long-term consequences of public measures. For example, one participant had been an active local politician earlier. She shared the story of how a recent development had split her neighborhood in half, and led to an escalating conflict regarding concerns about further expansion. Due to her former involvement as a local politician, did this participant have in-depth knowledge about regulation plans and other less-immediate aspects of the situation. She could thereby anticipate future consequences in a realistic way, which made her able to see long-term advantages where others saw threats. Moreover, it seems like getting de-facto access to decision-making processes at higher levels enabled inhabitants to establish shared understandings with those in charge, and thereby, to make compromises between personal preferences and public needs:

P: *“ Well, society has a lot of things they need to do- like building this culture-house over there. Well, it ruins our view. Not that the view was so fantastic before, but there was a free space, with soccer-games in the summer and ice-skating during wintertime- a fantastic place. We thought about where to protest, but we could not really do that either!”*

This might be an expression of the ability to re-define boundaries of meaningful areas-of-life, which is linked to a strong SOC ([20]: p.24). Moreover, being involved into decision-making processes increased acceptance of the municipality as a legitimate decision-maker (‘things are under control’), and the compromise did not threaten her strong sense of ownership towards her neighborhood [5].

#### Developing ownership and internalizing resources

Notions of perceived ownership became visible in all focus groups. Ownership was expressed through possessive attributions such as “*our house*”, “*my pier*”, “*my village*”. Perceived ownership influenced whether something was perceived as a resource: “*This house is health-promoting because you own it. It gives you something extra”*, as one participant puts it. This is supported by Cowling and Billings, claiming “*a sense of personal ownership (…) [was] all-important*” ([22]: p.998). Perceptions of ownership implied a sense of responsibility for the resource. Such perceptions are linked to “*commitment*” ([21]: p.116). A sense of ownership or commitment also triggered a variety of activities, including activities directed at the resource itself, such as shared cleaning-efforts. Such shared activities brought together various NGOs and inhabitants, and seem favorable for the establishment of shared understandings, broadening perspectives, and reinforce shared ownership of the neighborhood. Threats towards owned resources triggered action:

P: “*All of this, it has led me to really love my village. And if there is any threat to it, I have, you could say, developed a rather warrior-like mindset.”*

How inhabitants coped with perceived threats was heavily influenced by available resources, as illustrated above by the example of the participant mobilizing existing resources to influence a planned development. In contrast, neighborhood (A) experienced a similar threat, and perceived to understand the situation better than the central administration, but being excluded from decision-making. As existing resources seemed to fall short, they created their own channel-of-influence:

P: “*and then we had a kind of action-day against the “logistical junction”. We gathered people at the community-house, and had hired a bus. Then we marched towards the hotel carrying torches, to make the politicians come out and meet us»”*

Even if the impact of this action on the decision stays unknown, did participants describe the following experiences as positive: they perceived the process as both unifying and empowering. Shared perceptions of independence from the municipality were reinforced. By activating their social resources, this community was able to create a new, temporary structural resource. Might this ability build on experiences from creating, running and maintaining their own community-house?

Taken together, a sense of ownership over resources might imply that they are experienced as a part of oneself- with all the joys and responsibilities that implies. According to the above, does it seem reasonable to assume links between commitment, perceived ownership and active engagement with resources, all of which facilitate experiences touching upon all three dimensions of SOC [20]? Does this imply that they are important components of the internalization-process that turns external resources into GRRs [21]? In that case, providing neighborhood-resources that facilitate active engagement, commitment and the development of local ownership may be beneficial HP-strategies.

### Limitations and methodological issues

First, the focus of this study was on the individual, not collective SOC. Accordingly, unravelling individual and collective SOC was a major issue during the analysis. For example, are individual perceptions of being collectively independent from society an expression for individual or collective SOC? Antonovsky claimed that a strong group-SOC would facilitate for SOC-strengthening experiences and thereby benefit individual SOC [20]. Our findings support this by indicating that collective experiences can be generalized into individual SOC, and that a strong collective SOC facilitates for SOC-strengthening experiences (such as running a community house). Thus, collective SOC supports individual SOC by facilitating life events, and structuring their outcome.

On the other hand, the impact of individual SOC on collective SOC is less clear. According to Antonovsky would strong-SOC individuals seek affiliation with strong-SOC groups [20]. Intuitively, one might assume that strong-SOC individuals facilitate and structure collective experiences for their in-groups, and thereby strengthen group-SOC. However, findings suggest a more complex relationship: for example, in neighborhood C, participants would expressed a firm belief of being able to cope collectively. However, examples contradicted these assumptions. For example, residents would wish for internal collective transport. Even if ‘everyone’ agreed about the benefits of collective transportation, did most residents not consider it worthwhile to actually use the bus during a limited action-period. Instead, they chose individual coping strategies and mobilized private resources- thereby spoiling the chance to become collectively independent from such private resources. Maybe a strong individual SOC, combined with personal resources (a car and time to drive your kids) actually prevented the inhabitants from taking collective action? Is it possible that collective actions and resources, as all GRRs, are only activated when needed- that is, when personal resources fall short? This goes beyond the scope of this article. However, the above indicates that we cannot automatically assume that groups of strong-SOC individuals display a strong collective SOC.

#### Contextualising findings

Besides collective SOC, different coping strategies may be due to additional circumstances, such as historical references and collective identities. For example, the local identity in neighborhood A includes a strong working-class consciousness, and creating channels of influence through public protest might be a tried-and-true strategy. Contrarily, neighborhood C was newly established. Inhabitants failed attempt to take collective action might be due to a lack of shared experiences and historical references of doing so. One should be careful not to confuse collective SOC with other social resources such as social capital, which might have a stronger action-component [45].

Next, the context for this study is described as a resource-rich municipality with well-off inhabitants [46]. Findings from the population-survey suggest that the inhabitants have above-average strong SOC, and are highly satisfied with the availability and quality of their neighborhood- resources [18]. Additionally, it might be reasonable to assume that the inhabitants with weaker SOC are less inclined to participate in focus groups- this might even have reinforced elite-bias by describing the experiences of strong-SOC individuals [31]. This may lead to an underestimation of the impact of power-inequalities within neighborhoods [41], and limit the generalizability of findings onto less privileged settings or populations. Just as placing resources into a context might widen the gap in health [41], is it possible that facilitating active involvement and ownership favors already-strong-SOC individuals. Dominant groups might take charge over shared resources and thereby establish ownership at the expense of other groups, which might feel excluded and alienated.

This is supported by the notion that all focus groups discussed people not participating in the neighborhood. Such withdrawal was described as a personal choice which “*you have to respect*”. However, such descriptions were also repeatedly challenged by alternative explanations, including anxiousness for being of inconvenience, or by a lack of coherent information. As described in the context of information, something might be intended to be inclusive, but still feel excluding from the ‘outside’. This is a potentially interesting perspective, which nevertheless exceeds the scope of this paper. Taken together, described mechanisms might reflect experiences at ‘the upper side’ of SOC.

#### Analyzing focus-group discussions

There are some specific challenges linked to the analysis of focus-group-interviews [34]. First of all, including real-life groups might give insights into well-established understandings, but offer little insights into the processes linked to the establishment of such understandings [44]. However, even if participants were real-life neighbors, they did not represent a homogenous group. Additionally, including people from two approximate neighborhoods into one focus group indicates a heterogeneous sampling tactic. For the purposes of this study, this was considered beneficial to highlight the context-dependency of resources, and to stimulate the participants to discuss the processes that are linked to perceiving something as a resource across contexts.

Moreover, as a focus group represents a real-life experience for participants, information may have been altered, withheld or exaggerated to ensure social acceptance, or to prevent damage to neighborhood-relations [31]. On the other hand, gathering real-life neighbors did provide valuable insights into social processes and ongoing discussions within the community [34].

Including focus groups and in-depth interviews into one analysis might prove challenging. However, as the two types of interview are assigned to different parts of the analysis (develop versus confirm categories), including both is considered an advantage: it enabled us to achieve triangulation between data from neighborhood-based focus groups and from a more private setting. Moreover, in-depth interviews indicated data-saturation, as they did not provide substantial new information [31].

Last not least, specific challenges are linked to line-by-line coding, while simultaneously focusing on social interactions playing out over longer passages. However, one may wonder if the meaning of a particular line always emerges from its context. Moreover, as studying social processes is one of the most pronounced advantages of focus group design [34, 44], emerging information about social processes could not be dismissed. We applied tools from systemic analysis to meet these challenges, and grasp how singular utterances influence the developing focus-group-context and vice versa. Applying two levels-of-analysis provided interesting insights into how resources are identified, described and evaluated through social interactions. Simultaneously, applying two levels-of-analysis was beneficial for constant comparison and triangulation, as information from one level could be challenged, confirmed or explained by the other one [44].

### Future directions

With all the above considerations in mind, our findings seem to empirically support some of the less researched parts of Salutogenesis. Moreover, findings contribute to making Salutogenesis a beneficial HP-theory. First, a focus on every-day life is suggested, including everyday resources and -experiences. The daily hassles that Antonovsky dismissed by stating “*I see no way in which they can have any impact on the SOC*” ([20]: p.30) might be more important than anticipated. Everyday life and daily hassles seem important for identifying, potentiating and internalizing resources- which thereby become ready to be activated in times of major challenges. This is an advantage for HP-practitioners, as it enables them to focus on everyday-life and -settings.

#### Implications for practice

Findings suggest that health promoters should focus on the repeated experiences people make during everyday-life. Active engagement with, and perceived ownership of resources seem beneficial angles in this regard. Developing ownership over resources seems beneficial for SOC-strengthening experiences, and seems closely linked to active engagement at different levels of involvement. Moreover, findings suggest that intellectual meaning-making is a more active process than earlier anticipated. The establishment of stable channels of communication within and between neighborhoods (and municipalities) might prove beneficial for shared understandings, as well as broadening understanding of the sometimes conflicting needs municipalities must negotiate.

Two issues emerged in this context: first, matters of over- or underload regarding responsibility for resources: resources can become burdens. To avoid overload, municipalities should step in and sustain a certain level-of-maintenance in times of little interest, and know when to pull out and re-locate responsibility in the neighborhood. Next, to uncritical transfer power and responsibility into neighborhoods may reinforce internal inequalities, as strong-SOC individuals are more likely to recognize and take control over such resources. Thus, even inclusive strategies for planning, running and developing resources might unintentionally widen the gap in health unless applied critically [41]. A certain level-of-observation and clear guidelines might be beneficial. Moreover, direct efforts to include disadvantaged groups in all steps-of-development might prove beneficial, both in terms of improved relevance of resources, and in terms of facilitating for SOC-strengthening experiences. This might be a beneficial strategy to close the gap in health, as shared neighborhood-resources might be more important for people with fewer personal resources [5].

#### Theoretical considerations and implications for further research

The aim of this paper was to develop empirically grounded knowledge about how SOC can be strengthened through neighborhood-resources. According to recent research as well as Antonovsky’s own understandings [20], is this an important next step for developing Salutogenesis as a HP-theory. In addition to verifying large parts of Antonovsky’s original framework [20, 21], do our findings make substantial contributions to understanding the development of SOC.

First, findings suggest that everyday life, and especially everyday-challenges might contribute more to the development of SOC than previously anticipated. Internalization can be understood as making a variety of experiences with the resource that reveal why and how it works, and thereby become competent in flexible use and re-use of resources and strategies, including the identification of resources in unfamiliar settings [21]. This also implies a new understanding of ‘significant life events’ as experiences touching into comprehensibility, manageability and meaningfulness. A significant life event challenges learned coping strategies, offers possibilities to explore potential resources, and deepens understandings about why and how they are a resource. In other words, they are characterized by a (internal or external) reorganization of resources. This facilitates for the flexible strategies characterizing an authentic strong SOC ([20]: p.25).

Next, the insight that comprehensiveness is closely linked to active dialogue and meaning-making through power-equal social interactions might be a major contribution to theory-development. It might explain why the earlier research failed to confirm links between comprehensibility and clear, consistent and ordered messages [42]. This new understanding craves for more empirical validations, however, matters of power should be taken into account in the development of SOC-strengthening communication strategies.

Finally, all of the above has led to a new description of SOC as the ability to understand how and why resources work, including the ability to adapt, replace or re-define resources under shifting conditions. This also defines the point at which resources are fully internalized, and have become a GRR: when we understand the why and how. It also links internalization to coping strategies that can be assessed empirically, by the extent to which a given resource can be applied flexibly.

These insights imply a number of possibilities for future research: First, all of the new understandings and conceptualizations discussed above should become subject to further investigation, with a special focus on specialized and generalized resistance resources (SRR/GRR). Do they represent ‘active’ and ‘passive’ relationships with resources? A deeper look into these concepts may also yield insights into the internalization-process, and how it can be facilitated.

Moreover, efforts should be made to unravel individual and collective SOC, as well as collective SOC and social capital. All three appear to be valuable concepts within HP; however, to develop effective strategies, we should be able to define and distinguish them in a meaningful way. Promoting social capital in a neighborhood might require different strategies than strengthening collective SOC- yet the latter might enable the activation of the former.

Last but not least, to achieve inclusive planning and shared responsibility can be difficult. Thus, more research on inclusive planning strategies and health-promoting processes are needed to develop and systematize routines into working-models taking all these aspects into account [47].

## Conclusion

This study aimed at validating and critically examine some of the less researched parts of Salutogenesis: how the SOC is developed and strengthened through the internalization of external resources. Findings from focus groups and in-depth interviews suggest that the daily hassles and repeated experiences of every-day life might be a crucial arena for potentiation, and make experiences under shifting conditions which enable individuals to understand how and why resources work in a given setting. Active engagement with resources seem to facilitate for experiences touching into comprehensibility, manageability and meaningfulness, and might thereby be a favorable condition for internalization. Participation in power-equal, meaning-making dialogue can broaden perspectives by including other’s experiences in this understanding, and enable individuals to develop a more coherent picture of reality. Taken together, a new understanding emerges describing SOC as the ability to understand why and how resources work, and thereby, how they might be applicable in flexible ways. These insights make valuable contributions in developing Salutogenesis as a beneficial theory for HP-research and practice, as it links the SOC closer to every-day life and societal issues as implied in the SDoH and a HiAP-approach.

## Abbreviations

GRRs: Generalised Resistance Resources
HiAP: Health in all policies
HP: Health Promotion
SDoH: Social determinants of Health
SES: Sosio-economic status
SOC: Sense of coherence
SRRs: Specific Resistance Resources
WHO: World Health Organisation

## References

[1] WHO. The Ottawa-charter for Health Promotion*.* Ottawa: World health Organisation, 1986. http://www.who.int/healthpromotion/conferences/previous/ottawa/en/ Accessed 21 March 2017.

[2] WHO. Shanghai declaration on promoting health in the 2030 Agenda for sustainable development. Shanghai, World health Organisation. http://www.who.int/healthpromotion/conferences/9gchp/shanghai-declaration.pdf?ua=1. Accessed 06 March 2017. 10.1093/heapro/daw103 28180270

[3] Lindström B, Eriksson M (2012). From healthy settings to sustainable healthy societies: the salutogenic approach to planning and health promotion. World Health Design.

[4] Bloch P, Toft U, Reinbach HC, Clausen LT, Mikkelsen BE, Poulsen K, Jensen BB. Revitalizing the setting approach- supersettings for sustainable impact in community health promotion. Int J Behav Nutr Phys Act. 2014; doi:10.1186/s12966-014-0118-8. 10.1186/s12966-014-0118-8 PMC4172849 25218420

[5] Marmot M, Friel S, Bell R, Houweling T (2008). Taylor. Closing the gap in a generation: health equity through action son the social determinants of health. Lancet.

[6] WHO. Our cities, our health, our future: Acting on social determinants for health equity in urban settings. Kobe, Japan: Report to the WHO Commission on Social Determinants of Health from the Knowledge Network Urban Settings, 2007.

[7] WHO. Addressing the social determinants of health. The urban dimension and the role of local government*.* London, UK: WHO, Institute of Health Equity, University College of London, 2012.

[8] WHO. Environmental health inequities in Europe. Assessment report*.* Bonn, Germany: World Health Organisation, 2012.

[9] Kruize H, Droomers M, van Kamp I, Ruijsbroek A (2014). What causes environmental inequalities and related health effects? An analysis of evolving concepts. Int J Env Res Public Health.

[10] Abraham A, Sommerhalder K, Abel T (2010). Landscape and well-being: a scoping study on the health-promoting impact of outdoor environments. Int J Public Health.

[11] Angotti T. Towards the healthy city: People, places, and the politics of urban planning. Sci Society. 2013;77:595–597 (2).

[12] de Nazelle A, Nieuwenhuijsen MJ, Anto JM, Brauer M, Briggs D, Braun-Fahrlander C, Cavill N, Cooper AR, Desqueyroux H, Fruin S, Hoek G, Panis LI, Janssen N, Jerrett M, Joffe M, Andersen ZJ, van Kempen E, Kingham S, Kubesch N, Leyden KM, Marshall JD, Matamala J, Mellios G, Mendez M, Nassif H, Ogilvie D, Peiró R, Pérez K, Rabl A, Ragettli M, Rodríguez D, Rojas D, Ruiz P, Sallis JF, Terwoert J, Toussaint JF, Tuomisto J, Zuurbier M, Lebret E (2011). Improving health through policies that promote active travel: A review of evidence to support integrated health impact assessment. Environ Int.

[13] Faskunger J (2013). Promoting active living in healthy cities of Europe. J Urban Health.

[14] Frumkin H (2003). Healthy places: exploring the evidence. Am J Public Health.

[15] Gidlow C, Cochrane T, Davey RC, Smith G, Fairburn J (2010). Relative importance of physical and social aspects of perceived neighbourhood environment for self-reported health. Prev Med.

[16] Lachowycz K, Jones AP (2013). Towards a better understanding of the relationship between greenspace and health: development of a theoretical framework. Landscape Urban Plan.

[17] Maass R, Espnes GA, Lillefjell M, Mittelmark MB, Sagy S, Eriksson M, Bauer G, Pelikan JM, Lindstroem B, Espnes GA (2017). The application of Salutogenesis in cities and towns. The handbook of Salutogenesis.

[18] Maass R, Lindström B, Lillfjell M (2014). Exploring the relationships between perceptions of neighborhood-resources, sense of coherence and health in a Norwegian neighborhood. J Public Health Res..

[19] Maass R, Kloeckner CA, Lindström B, Lillefjell M (2016). The impact of neighborhood social capital on life satisfaction and self-rated health: a possible pathway for health promotion?. Health Place..

[20] Antonovsky A (1987). Unravelling the mystery of health.

[21] Antonovsky A (1979). Health, stress and coping.

[22] Cowley S, Billings JR (1999). Resources revisited: Salutogenesis from a lay perspective. J Adv Nurs.

[23] Apers S, Rassart J, Luyckx K, Oris L, Goossens E, Budts W, Moons P. Bringing Antonovsky’s salutogenic theory to life: a qualitative inquiry into the experiences of young people with congenital heart disease. Int J Qual Stud health Well-being. 2016; doi:10.3402/qhw.v11.29346. 10.3402/qhw.v11.29346 PMC4778384 26942908

[24] Eriksson M, Lindström B (2006). Antonovsky’s Sense of coherence and it’s relation with health: a systematic review. J Epidemiol Commun H..

[25] Eriksson M, Lindström B (2007). Antonovsky’s Sense of coherence and it’s relation with quality of life: a systematic review. J Epidemiol Commun H..

[26] Rivera F, Garica-Moya I, Moreno C, Ramos P. Developmental contexts and sense of coherence in adolescence: a systematic review. J Health Psychol. 2013; doi:10.1177/1359105312455077. 10.1177/1359105312455077 22947890

[27] Marsh SC, Clinkinbeard SS, Thomas RM, Evans WP (2007). Risk and protective factors predictive of sense of coherence during adolescence. J Health Psychol.

[28] Nash JK (2002). Neighborhood effects on sense of school coherence and educational behavior in students at risk of school failure. Child Sch.

[29] Lillefjell M, Maass R, Espnes GA (2013). Helse og livskvalitet i Malvik Kommune. Rapport 01/2013.

[30] Constructing CK, Theory G (2006). A practical guide through qualitative analysis.

[31] Patton MQ (2002). Qualitative research and evaluation methods.

[32] Freeman T. ‘Best practice’ in focus group research: making sense of different views. J Adv Nurs. 2006:491–7. 10.1111/j.1365-2648.2006.04043.x 17078825

[33] Kitzinger J. Qualitative research: introducing focus groups. BMJ**.** 1995;311:299-302 10.1136/bmj.311.7000.299 PMC2550365 7633241

[34] Kitzinger J (1994). The methodology of focus groups: the importance of interaction between research participants. Sociol Health Ill.

[35] Rotter JB. Generalized expectancies for internal versus external control of reinforcement. Psychol Monogr- Gen A. 1966; doi:10.1037/h0092976. 5340840

[36] Bull T, Mittelmark M, Kanyeka NE (2013). Assets for women living in deep poverty: through a salutogenic looking-glass. Crit Public Health.

[37] Eriksson M, Lindström B. Validity of sense of coherence scale: a systematic review. J Epidemiol Community Health. 2005:460–6. 10.1136/jech.2003.018085 PMC1757043 15911640

[38] Hakanen JJ, Feldt T, Leskinen E (2006). Change and stability of sense of coherence in adulthood: longitudinal evidence from the healthy child study. J Res Pers.

[39] Feldt T, Leskinen E, Koskenvuo M, Suominen S, Vahtera J, Kivimaeki M (2011). Development of sense of coherence in adulthood: a person-centered approach. The population-based HeSSup cohort study. Qual Life Res.

[40] Ville I, Khlat M (2007). Meaning and coherence of self and health: an approach based on narratives of life events. Soc Sci Med.

[41] Mittelmark MB. Unintended effects in settings-based health promotion.Scand J Public Health. 2014. Doi:10.1177/1403494814545108 10.1177/1403494814545108 25416569

[42] Sagy S, Antonovsky A (2000). The development of the sense of coherence: a retrospective study of early life experiences in the family. Int J Aging Hum Dev.

[43] Csikszentmihályi M. Flow: the psychology of optimal experience*.* New York, Harper & Row ISBN 978-0-06-016253-5.

[44] Morgan D.L. & Krueger R.A. (1993) When to use focus groups and why. In (Morgan, D. (Ed) successful focus groups: advancing the state of the art*.* London, Sage. p.3–19.

[45] Eriksson M. Social capital and health- implications for health promotion. Glob Health Action. 2011; doi:10.3402/gha.v4i0.5611. 10.3402/gha.v4i0.5611 PMC3036711 21311607

[46] Folkehelseinsituttet. Folkehelseprofil 2016 Malvik http://khp.fhi.no/PDFVindu.aspx?Nr=1663&sp=1&PDFAar=2016. Accessed 20 March 2017.

[47] Lillefjell M, Wist G, Magnus E, Anthun K.S, Horghagen S, Espnes GA, Knudtsen MS. Trøndelagsmodellen for folkehelsearbeid. Rapport 01/2017, ISBN 978–82–93158-34-9, ISSN 1892–6207. Trondheim: Rapportserie fra NTNU Senter for helsefremmende forskning; 2017.

